# Aerially Applied Zinc Oxide Nanoparticle Affects Reproductive Components and Seed Quality in Fully Grown Bean Plants (*Phaseolus vulgaris* L.)

**DOI:** 10.3389/fpls.2021.808141

**Published:** 2022-01-12

**Authors:** Hajar Salehi, Abdolkarim Chehregani Rad, Hamidreza Sharifan, Ali Raza, Rajeev K. Varshney

**Affiliations:** ^1^Laboratory of Plant Cell Biology, Department of Biology, Bu-Ali Sina University, Hamedan, Iran; ^2^Department of Natural Science, Albany State University, Albany, GA, United States; ^3^Key Laboratory of Ministry of Education for Genetics, Breeding and Multiple Utilization of Crops, Oil Crops Research Institute, Center of Legume Crop Genetics and Systems Biology, College of Agriculture, Fujian Agriculture and Forestry University, Fuzhou, China; ^4^Center of Excellence in Genomics and Systems Biology, International Crops Research Institute for the Semi-Arid Tropics (ICRISAT), Hyderabad, India; ^5^State Agricultural Biotechnology Centre, Centre for Crop and Food Innovation, Murdoch University, Murdoch, WA, Australia

**Keywords:** reproductive components, pollen dysfunction, seed productivity, food quality, nanotechnology

## Abstract

The development of reproductive components in plant species is susceptible to environmental stresses. The extensive application of zinc oxide nanoparticles (nZnO) in various agro-industrial processes has jeopardized the performance and functionality of plants. To understand the response of the developmental (gametogenesis and sporogenesis) processes to nanoparticles (NPs) exposure, the aerial application of nZnO and their ionic counterpart of ZnSO_4_ at four different levels were examined on bean plants (*Phaseolus vulgaris*) before the flowering stage. To evaluate the mentioned processes, briefly, flowers in multiple sizes were fixed in paraffin, followed by sectioning and optical analysis. The possibility of alteration in reproductive cells was thoroughly analyzed using both light and electron microscopes. Overall, our results revealed the histological defects in male and female reproductive systems of mature plants depend on NPs levels. Furthermore, NPs caused tapetum abnormalities, aberrations in carbohydrate accumulation, and apoptosis. The nZnO induced abnormal alterations right after meiosis and partly hindered the microspore development, leading to infertile pollens. The seed yield and dry weight were reduced to 70 and 82% at 2,000 mg L^–1^ nZnO foliar exposure, respectively. The sodium dodecyl sulfate (SDS)-polyacrylamide gel electrophoresis pattern showed the increased expression of two proteins at the molecular weight of 28 and 42 kDa at various concentrations of nZnO and ZnSO_4_. Overall, our results provided novel insights into the negative effect of nano-scaled Zn on the differential mechanism involved in the reproductive stage of the plants compared with salt form.

## Introduction

Life cycle of flowering plants (angiosperms) translates into two generations of sporophyte and gametophyte, in which a haploid gametophyte (i.e., pollen grain and embryo sac) is formed from a diploid sporophyte (Yadegari and Drews, 2004). Due to the increasing trend of environmental pollution, the intrusion of contaminants in the plant reproductive life cycle (i.e., generative phase and gametophyte) is inevitable (Zinn et al., 2010; De Storme and Geelen, 2014; Paupière et al., 2014; Salehi et al., 2021a). Overwhelming studies have reported that the sexual reproduction system and seed productivity are severely affected by environmental stresses (Zinn et al., 2010; De Storme and Geelen, 2014; Paupière et al., 2014; Salehi et al., 2021a). For example, severe reproductive effects including disproportionate time intervals of gametophyte development, sporophytic anomalies, and cellular damages of gametes have been reported under temperature stress (Zinn et al., 2010). These alterations are difficult to track due to the short course of gametogenesis and fertilization within sporophytic tissues. Therefore, there is a lack of knowledge in the developmental phase to understand the biological response of the plant reproductive system under normal and stress conditions.

In modern agricultural practices, nanotechnology advancement has developed as a revolutionary breakthrough in food science and engineering. Nanomaterials are vital components of many achievements in agricultural food safety. Nanoparticles (NPs) have immense potential to develop highly precise and effective crop cultivation techniques. Zinc oxide NPs (hereafter named nZnO) possess unique properties, such as high surface area and adsorption capacity, easy operation, and cost-effective production (Khan et al., 2019). The applications of nZnO have been found to stimulate plant growth, chlorophyll synthesis, and nutritional fortification. For instance, some recent studies have shown the beneficial effects of nZnO on seed germination, shoot, and root growth in different plants including maize (López-Moreno et al., 2017) and wheat (Awasthi et al., 2017). Another recent study showed that cilantro (*Coriandrum sativum* L.) grown in soil amended with nZnO (400 mg kg^–1^) promotes defense responses through improved photosynthetic pigments such as chlorophyll and decreased lipid peroxidation contents (Pullagurala et al., 2018). Recently, Dimkpa et al. (2019) reported that nZnO (up to 5 mg kg^–1^) mitigate the adverse effect of drought stress in sorghum (*Sorghum bicolor* L.), which improved the total potassium acquisition and enhanced grain productivity. However, the phytotoxicological effects of nZnO on mature plants are highly neglected in the literature (Chen et al., 2016; Jahan et al., 2018; Singh et al., 2018). Impacts of foliar application of NPs have demonstrated a positive correlation with applied concentrations. However, the presence of nZnO associated with the release of reactive oxygen species (ROS) is a likely reason for inhibitory effects on plant performance (Zhao et al., 2013; Bandyopadhyay et al., 2015; Wang et al., 2016). The extent of ROS release depends on several factors, including NPs size and surface charge, plant species, and exposure time (Marslin et al., 2017). The applied NPs concentrations may expose potent effects that dominate all other effects induced by different environmental factors or by triggering them. Therefore, we hypothesized that the perturbation of pollen and embryo sac cells is due to growth inhibition caused by oxidative stresses and the negative effects of NPs on the plant reproduction system. To our best knowledge, there is no study on the consequences of nZnO on the reproductive phase of plant development, mainly in bean. In this study, we applied nZnO at four different levels to elucidate their effects on the reproductive phase and seed productivity of bean (*Phaseolus vulgaris* L.) plants. The main objective of this study was to identify the potential impacts of nZnO on reproductive organs, including pollen and embryo sac.

## Materials and Methods

### Materials

The nZnO (10–30 nm 99% purity) was purchased from Nanosany Company (US-Nano), Mashhad, Iran. The NPs were fully characterized using transmission electron microscopy (TEM) (100 kV Philips, EM208, Sigma-Aldrich, Germany) and scanning electron microscopy (SEM) (JSM-840A, JEOL, Tokyo, Japan) images. Briefly, the specific surface area ranged from 20 to 60 m^2^ g^–1^ with a density of 5.6 g/cm^3^. A detailed analysis of NPs physiochemical properties is provided in Supplementary Figure 1. Zinc sulfate (ZnSO_4_.7H_2_O) was supplied by Sigma-Aldrich (Milan, Italy). The formaldehyde, glacial acetic acid, ethanol, acetocarmine, acid fuchsin, and orange G used at analytical grade were purchased from Sigma-Aldrich (Heidelberg, Germany).

### Experimental Design

Pinto bean (*P. vulgaris*) seeds were purchased from Behineh Sazane Sabze Mehregan Company (Biotek Seeds, Iran). Approximately 100 seeds were sterilized in 5% sodium hypochlorite for 15 min and germinated in moist sand. After 4 days of germination, healthy seedlings were transplanted in pots (25 cm × 30 cm) containing local soil (20% clay, 10.6% silt, 69.4% sand, 2.3% organic matter, and pH 7.6). The growth condition was controlled in a greenhouse under a 16/8 day-night cycle at 30/16°C, and the average humidity was 70%. Among 3-week-old plants (Biologische Bundesanstalt, Bundessortenamt und CHemische Industrie (BBCH) 13-15; phenological stage: 3–5 true leaves), healthy plants were selected for further nZnO foliar treatments.

### Foliar Application of nZnO

Foliar solutions of nZnO were prepared in four concentrations (250, 500, 1,000, and 2,000 mg L^–1^) in ultrapure water (18Ω). The nZnO concentrations were selected based on previous studies reporting contradictory findings of their function in terms of both phytotoxicity and positive effectiveness (Torabian et al., 2016; García-Gómez et al., 2018; García-López et al., 2019). The nZnO dispersion was subjected to 30 min sonic bath at 25°C to ensure the homogenous dispersion of the NPs. As a positive control for the nZnO, the zinc sulfate (ZnSO_4_) solution (normally applied as a foliar spray to the crop) at the aforementioned levels was prepared. All pots were divided into four groups with five replicates for each concentration, and the soil surface was thoroughly covered with plastic films to avoid exposure of NPs. The suspensions of NPs were manually sprayed on the leaves every 2 days at 9 a.m. for 2 weeks. A total volume of 200 ml of each concentration of either nZnO or ZnSO_4_ was applied foliar. Next, treatments were subjected to air drying, and the pots were kept in the greenhouse under the aforementioned conditions (16/8 day-night cycle at 30/16°C, and the average humidity of 70%). The irrigation with tap water took place every 2 days until the termination stage.

### Sample Harvest and Developmental Studies

At the termination stage, flowers through the growth transformation from young buds to mature flowers and pods were carefully collected with a blade. Immediately, the samples were stabilized in formalin aceto-alcohol (FAA_70_ composed of 3% formaldehyde, glacial acetic acid, and 70% ethanol at 2:1:17 proportions, respectively) for 24 h and were rinsed with deionized water five times to remove residual of the FAA solution and stored in 70% ethanol for further developmental analysis. The plant tissues were subjected to hydration in exposure to ethanol gradual series (70, 80, 90, and two times in 100%) and finally toluene. The hydrated tissues were placed into liquid paraffin and incubated in an oven at 65°C for 3 days. The embedded tissues in paraffin were cut to slices of ∼8.5 μm thickness using a rotary microtome (Micro DS 4055, Germany) and then fixed onto glass slides. The combination of hematoxylin and eosine solutions were used for staining internal organelles (Chehregani Rad and Salehi, 2016). The prepared slides were quantified using the optical microscope (LABOMED model LX50, Germany), and dynamic changes in male and female gametophytes were captured using LABOMED digital camera (Germany). At least ten samples of each developmental stage per pot and over 100 experimental slides were observed to confirm the possible changes compared with controls.

### Pollen Viability Assay

To investigate the pollen viability, pollen and anthers were stained with acetocarmine (1% in acetic acid) and Alexander stains. Briefly, acetocarmine stained the viable pollens with regular shapes in reddish color, whereas non-viable pollen was pale. Alexander stain contained a mixture of 10 ml 96% ethanol, 10 mg malachite green, 50 ml distilled water, 25 ml glycerol, 5 g phenol, 5 g chloral hydrate, 50 mg acid fuchsin, 5 mg orange G, and 4% glacial acetic acid. For sample preparation, the dissected flowers that were about to open were selected and all tissues were removed except for anthers and then were placed into a 20 μl volume of Alexander staining solution on a microscopic slide and incubated for 3 h. After staining with Alexander, red or pink colors represented the viable pollen while the green-pale ones were non-viable. In the case of pollen viability, at least 20 slides per treatment were monitored. For additional characteristics, SEM (JSM-840A, JEOL, Tokyo, Japan) observation was carried out using a gold coating of dried pollen grains.

### Measuring the Seed-Related Traits and Zn Accumulation

The final measurements on seeds were performed at the end of the life cycle of bean plants (120 days after planting). Briefly, the number of pods for each cultivar, the total number of produced seeds per plant and per pod, and seed dry weight were evaluated at the termination stage. The total Zn content in seeds was also quantified using atomic absorption spectrometry (Varian Inc, Applied Science Co., Iran), following our previously published work (Salehi et al., 2021b). Dry seeds were gently washed with distilled water to remove any potential contamination. The oven-dried seeds were mechanically grounded using a grinding machine. Approximately 1 g subsample of each treatment was weighted for further acid digestion. A mixture of 65% HNO_3_ and 20% H_2_O_2_ at 5:1 proportion was used to digest the samples at 60°C for about 6 h. Samples were allowed to cool down to room temperature, and then each sample was diluted to 50 ml with deionized water and filtered using Whatman No. 42 filter paper (2.5 μM).

### Total Protein Content and Sodium Dodecyl Sulfate-Polyacrylamide Gel Electrophoresis

Bradford assay was used to determine total protein content (Bradford, 1976). Initially, seeds were grounded in liquid nitrogen until fully homogenized. The total protein was extracted using 25 mM phosphate buffer (KH_2_PO_4_) at 4°C. After centrifuge, the supernatants were used to measure protein concentration using UV/Vis Spectrometer (Biowave II, England) at 595 nm.

In addition, the sodium dodecyl sulfate-polyacrylamide gel electrophoresis (SDS-PAGE) pattern of bean seed proteins was performed using the extracted total protein. Briefly, the seeds were grounded using a mixer machine, and then total protein was extracted using sodium phosphate buffer (pH = 7) at 4°C. The Bradford standard assay was used to determine total protein content at 595 nm. The SDS-PAGE analysis was performed as follows: an equal proportion of buffer was loaded (0.125 M Tris-HCl, 4% SDS, 20% glycerol, 10% mercaptoethanol, and 0.1% bromophenol blue) and protein extract was boiled at 100°C for 5 min, and then loaded into 12% polyacrylamide gel holes, and finally, electrophoresis was run at 90 V (constant voltage) (Salehi et al., 2013). Coomassie Brilliant Blue R-250 (Sigma-Aldrich, Germany) was used for gel staining. To compare the molecular weight, an SDS-PAGE protein marker was also loaded (CinnaGen, Iran).

### Statistical Analysis

Data on the pollen irregularity, seed weight, and seed yield were analyzed using a one-way ANOVA followed by the Duncan test at *p* ≤ 0.05 using PASW Statistics 18.0 (SPSS Inc., Chicago, IL, United States). The statistical differences between datasets of each treatment were processed by *post hoc* test on a mean basis. All experiments were performed in five replicates.

## Results

### The Effect of nZnO and ZnSO_4_ on the Male (Anther and Pollen Grain) Development

**FIGURE 1:**
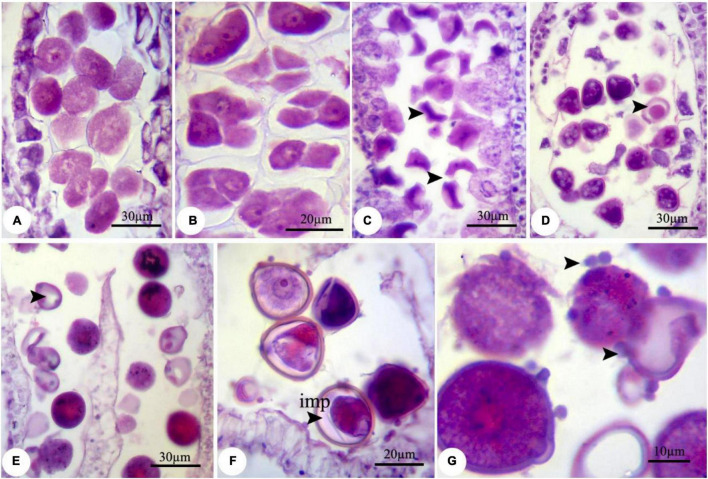
Observed modifications of the microsporogenesis process in bean plants exposed to 250 mg L^–1^ nZnO. **(A)** Pollen mother cells in the prophase stage with varied sizes. **(B)** The irregular placing of microspores in tetrad and degenerating some of them. **(C)** Released microspores have unusual sizes (arrows), microspores, and cytoplasmic content have degenerated. **(D)** Pollen sac containing microspores with early degenerated cytoplasm. **(E)** Full cytoplasmic-degenerated and paled microspores (arrow). **(F,G)** Off-key microspores and emerging spherical bodies on exine surface of pollen grain wall (arrows). imp, immature pollen grain.

During the nZnO and ZnSO_4_ exposure, no structural abnormalities were observed in anther shape and microsporocyte stage. However, from onset meiosis, unprecedented changes were realized. For example, reproductive structures were altered in both treatments, while the effects of nZnO were more prominent compared with other treatments; cellular dysfunction started at 250 mg L^–1^ of nZnO. The developmental stage was affected differently for each treatment depending on the applied doses. Under nZnO treatments, the initial phases of meiosis were affected, as at prophase I, the pollen mother cells (PMC) had various sizes, with some being degenerated (Figure 1A) compared with control plants (Supplementary Figure 2). Similarly, deformation of tetrads was observed at the tetrad stage, including the triads (languishing one of the cells) and irregular shapes (Figure 1B). In addition to tetrad irregularity, asynchronous in cytokinesis and wall formation in tetrads were visible. The released immature microspores were irregular and dominated by falciform or starlike (asteroid) (Figure 1C).

**FIGURE 2:**
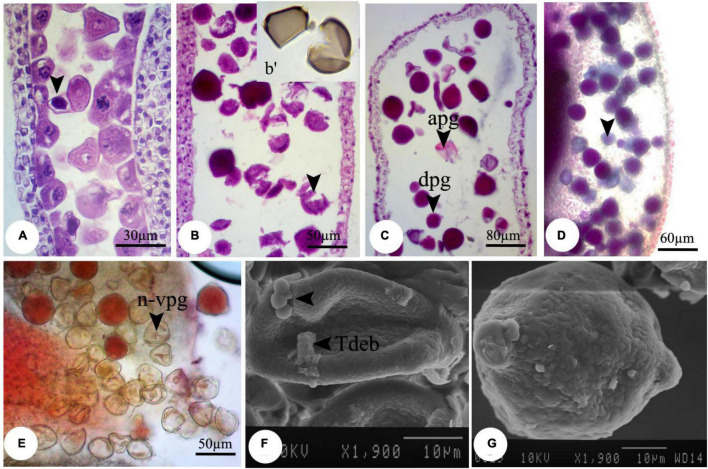
Observed modifications of the microsporogenesis process in bean plants exposed to 2,000 mg L^–1^ nZnO. **(A)** Anther loculus contains immature microspores with different shapes; some have completely degenerated, and others have been crumpled and plasmolyzed (arrow). **(B,b′)** Brittle mature pollen grains. **(C)** Irregular mature pollen grains with different shapes. **(D)** Alexander staining of anther, viable pollen grains (arrow). **(E)** Stained pollen grains by acetocarmine. **(F)** SEM image of the immature pollen grain and cytoplasmic exudation on the pollen surface is observed. **(G)** Mature pollen grain with an uneven surface. apg, abrasived pollen grain; dpg, degenerated pollen grain; n-vpg, non-viable pollen grain; Tdeb, tapetum debris.

Despite the anther and microspore growth, the cytoplasmic content of microspores was degenerated (Figures 1D,E), leading to form amorphous and likely non-viable pollen grains, which were more evident at higher concentrations. There were some spherical, bubble-shaped bodies on the pollen grains surface, which seemed to be the cytoplasmic exudations (Figures 1F,G). At high concentrations (especially 2,000 mg L^–1^), in most of the microspores, the nucleus and cytoplasm contents were plasmolyzed and agglomerated (Figure 2A). The other remarkable point was the premature disappearance of the tapetal cells in some pollen sacs (Figure 2B) or abnormally expanded and persisted tapetum cells in some others (Figure 2A), which may be leading to the disproportionate maturation of the microspores due to nutritional deficiency. Followed by pollen grains development, they were crushed and broken (Figure 2B,b′), likely due to defects in wall formation, allowing the cytoplasmic exudations and probably producing non-functional pollen grains (Figure 2C).

**FIGURE 3:**
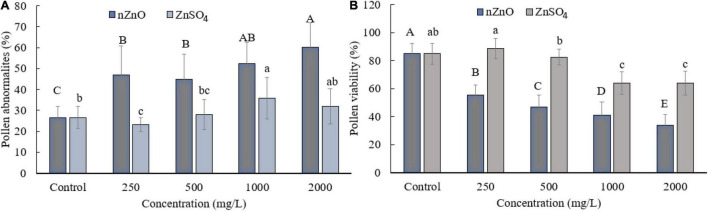
Grain abnormality rate of pollen **(A)** and viability **(B)** at different concentrations of nZnO and ZnSO_4_. The data are the average. Error bars represent mean ± SD. Different letters above the bar indicate the significant difference at *p* < 0.05.

**FIGURE 4:**
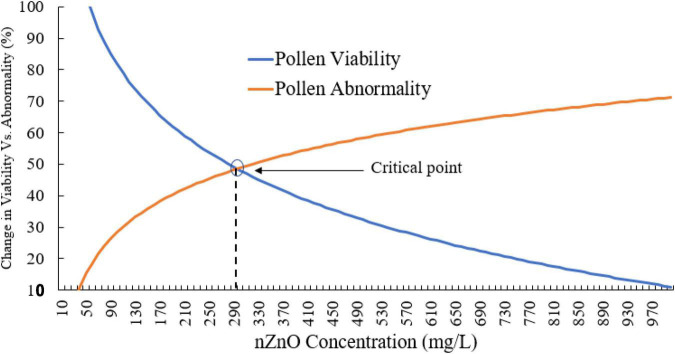
The simulated behavior of both pollen viability and abnormality is based on experimental values. The critical points indicate the limiting doses of the nZnO that will lead to phytotoxicity effects.

Moreover, compared with control plants (Supplementary Figure 3), in the highest dosage of nZnO, pollen grain ornamentations and its surface were bumpy, and SEM images showed some spherical-shaped bodies on the surface (Figures 2F,G). Pollen viability was also considered to assess the male gametophyte developmental stage and particularly the fitness of reproductive organs. Staining with Alexander and acetocarmine showed most pollen grains have lost their coloring ability and were non-viable (Figures 2D,E) in comparison with pollen grains in control plants (Supplementary Figure 3). Interestingly, approximately 61% of mature pollen grains were found in irregular and abnormal structures when exposed to 2,000 mg L^–1^ of nZnO (Figure 3). Figure 4 was derived from simulating the behavior of pollen abnormality and viability in exposure to a different range of nZnO.

The critical point can be seen in a range of 250–350 mg L^–1^ nZnO, which limits the pollen viability of the reproduction system. The pollen viability might be affected during different stages of the male development from early phases in anther to placing on the stigma. In this context, the low viability of pollens in nZnO treatment might be related to high dehydration and alternation in carbohydrates type and content.

**FIGURE 5:**
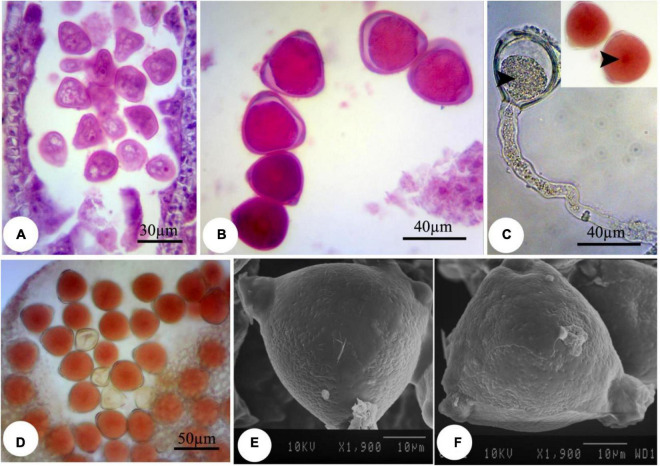
Induced changes in the microsporogenesis process in bean plants exposed to 2,000 mg L^–1^ ZnSO_4_. **(A)** Immature pollen grain. **(B)** Mature pollen grain. **(C)** Pollen grain along with pollen tube and its content. The central part of the pollen grain is more colorful (arrow). **(D)** Pollen grains staining with acetocarmine. **(E,F)** Scanning electron micrographs of the mature pollen grain.

Compared with nZnO, the effects of Zn ions on the deformation of male gametophyte development were insignificant (Figures 5A–F). However, further image analysis revealed that the cytoplasm tends to be more compressed in exposure to Zn ions compared with control plants, with a visible space between the cell wall and cytoplasmic content (Figures 5B,C). This observation was consistent with cellular acetocarmine staining (Figures 5B–D), which could be due to cytoplasm plasmolysis. However, no visible changes were observed in the pollen grains appearance compared with control plants using SEM images (Figures 5E,F).

### Female (Ovule and Embryo Sac) Development Under nZnO and ZnSO_4_ Exposure

**FIGURE 6:**
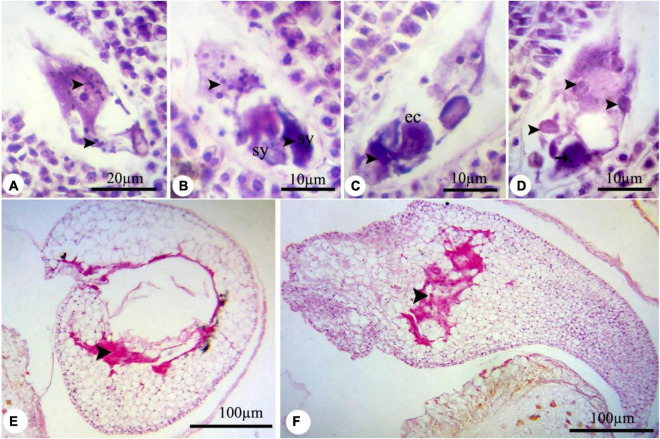
The nZnO-induced modifications of *P. vulgaris* female gametophyte at different concentrations. **(A)** The apoptosis of egg apparatus (arrows). **(B)** Apoptosis of central cell (arrow) and degenerating of the nucleus, and excessive coloration of synergids. **(C,D)** Abnormal division of endosperm and degenerating of nuclei. **(E,F)** Destruction of embryonic tissue (arrow) and irregularity in placing of cells. sy, synergid; cc, central cell.

In all treated plants, the female development responses were identical with the control plants (Supplementary Figures 4–6) until the functional megaspore division stage. The major alteration was observed at the eight-cell embryo sac stage. High concentrations of nZnO caused apoptosis of the oocyte apparatus (Figure 6A) and central cells (Figure 6B) as cellular pieces of degeneration processes were found in the embryo sac cytoplasm. Also, synergid cells seemed to bear serious damage in some ovules, resulting in their dysfunctionality (Figure 6C). Synergid cells govern pollen tube conducting, which such abiotic stresses may disturb this action and cause pollen grain infertility (Leshem et al., 2013). Furthermore, the induction of abnormalities in the endosperm division (Figure 6D) was noticed by dislocation and scattering of the nuclei of the nuclear endosperm in the cytoplasm. Compared with control plants (Supplementary Figure 6), embryo abortion occurred in most of the ovules at higher concentrations of nZnO (Figures 6E,F). Also, the interruption of embryonic tissue at different stages is likely due to cellular damage and fragility.

### The Evaluation of Seed Productivity at the End of the Plant Growth Cycle

**FIGURE 7:**
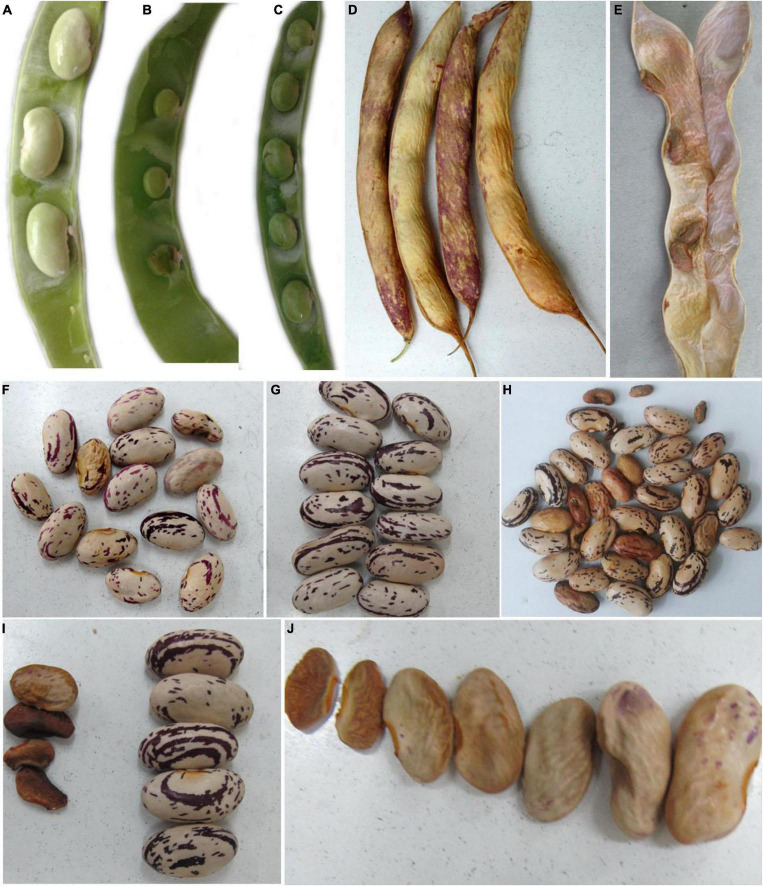
The images of sheath and seeds of treated and control plants. The sheath of control plant **(A,D)**, 2,000 mg L^–1^ nZnO **(B,E)**, and ZnSO_4_ **(C)**. Furthermore, **(F–H)** are related to the control seeds, 250 mg L^–1^ ZnSO_4_, and nZnO treatments, respectively. The characters of **(I,J)** represent seeds of plants exposed to 2,000 mg L^–1^ ZnSO_4_ and nZnO treatments, respectively.

The seed-related parameters upon exposure to nZnO and ZnSO_4_ are shown in Table 1. The corresponding images of pods and seeds are illustrated in Supplementary Figure 6. As shown in Table 1, both nZnO and Zn ions significantly altered the yield and weight of seeds (*P* ≤ 0.005). As expected, in exposure to 2,000 mg L^–1^, most of the ovules failed to transform into seeds. A negative correlation between the number of seeds and their productivity with nZnO levels was observed as approximately 70% of total seed reduction occurred at 2,000 mg L^–1^, compared with control plants. Similarly, the weight of the total seeds was reduced by 82% at the highest nZnO level. Lower numbers of pods (6.60 compared with 13.3 in control plants) and affected seed physiology may be caused by smaller plant elongation and increased flower abscission rate during growth term. The microscopic images revealed that seed development and growth were inhibited at the early stages. Two mechanisms may be responsible for this phenomenon: first, the potential lack of successive divisions of endosperm as the embryonic nutritive tissue and, second, impairing the cellular tissue of the embryo. Notably, both mechanisms lead to the inhibition of the embryo and the development of cotyledons. It is worth mentioning that plants showed different responses to Zn ions treatment in the same concentrations. For example, exposure to 250 mg L^–1^ increased the number of pods per plant (by 12%), the number of seeds per plant (by 31%), and seed dry weight (by 22%) in comparison with control plants (Table 1). Respectively, the appearance of the seed improved, and the lowest mortality rate was recorded (Figure 7). However, at 2,000 mg L^–1^ ZnSO_4_, the total yield and dry weight were insignificantly reduced by 12% and 11%, respectively.

**TABLE 1:** The comparison of Zn content and seed-related parameters of bean plants exposed to different concentrations of nZnO and ZnSO_4_ at the end of the growth period.

	Zn content (mg kg^–1^)	Pods per plant	Seeds per pod	Seeds per plant	Seed dry weight (g/10 seeds)
**nZnO (mg L^–1^)**					
0	45.5 ± 1.5*c*	13.3 ± 1.63*a*	5.2 ± 1.03*a*	67 ± 6.2*a*	3.19 ± 0.19*a*
250	52.3 ± 1.6*b*	12.0 ± 0.94*ab*	4.7 ± 0.94*ab*	55 ± 11.1*b*	2.60 ± 0.18*b*
500	58.9 ± 1.3*a*	12.1 ± 0.99*ab*	4.5 ± 0.84*ab*	53.6 ± 9.5*b*	2.07 ± 0.11*c*
1000	59.8 ± 0.4*a*	11.5 ± 2.27*b*	4.2 ± 0.91*bc*	47.3 ± 4.0*b*	1.68 ± 0.10*d*
2000	60.7 ± 0.3*a*	6.60 ± 1.83*c*	3.6 ± 0.69*c*	20.3 ± 2.5*a*	0.97 ± 0.11*e*
**ZnSO_4_ (mg L^–1^)**					
0	45.5 ± 1.5*a*	13.3 ± 1.63*bc*	5.2 ± 1.03*a*	67 ± 6.2*b*	3.19 ± 0.19*b*
250	48.6 ± 1.4*b*	14.9 ± 1.28*a*	5.3 ± 0.94*a*	88.3 ± 4.1*d*	3.89 ± 0.30*a*
500	50.3 ± 1.6*b*	13.2 ± 1.68*bc*	5.2 ± 1.03*a*	65.6 ± 10.4*c*	3.13 ± 0.27*b*
1000	55.2 ± 0.1*c*	13.5 ± 1.26*b*	4.8 ± 1.13*a*	67.6 ± 6.0*b*	3.05 ± 0.08*b*
2000	57.3 ± 0.9*c*	12.0 ± 1.15*c*	4.6 ± 0.51*a*	58.3 ± 7.0*a*	2.83 ± 0.30*b*

*Data represent mean ± SD. Different letters indicate statistical significance at p < 0.05.*

### The Sodium Dodecyl Sulfate-Polyacrylamide Gel Electrophoresis Profile of Seeds

**FIGURE 8:**
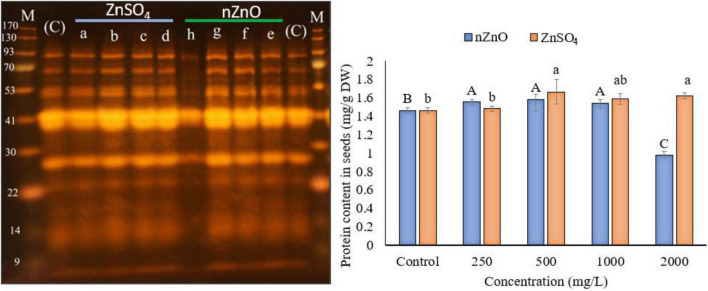
The left image presents the SDS-PAGE analysis, where the a–d indicated the cultivar genome in treatments exposed to 250, 500, 1,000, and 2,000 ZnSO_4_ (mg L^–1^), and e–h for 250, 500, 1,000, and 2,000 nZnO (mg L^–1^), respectively. The letters (C) and (M) indicate the control and marker plants, respectively. The plot on the right shows the protein content of dry seeds. The character above each bar indicates the statistical significance (*p* < 0.05).

To investigate the effect of applied nZnO and Zn ions on protein content, the total protein was measured, followed by SDS-PAGE analysis. Figure 8 shows a positive relationship between protein synthesis of the plant and an increasing dose of nZnO up to 1,000 mg L^–1^ compared with the control plants. The remarkable increase in width and intensity of 28 kDa protein band (Figure 8) may correspond to the pod storage protein (PSP), which acts as nutrient storage in plants exposed to stress. In agreement with this, we found the upregulation of PSP in leaves exposed to nZnO treatments compared with control plants as addressed in proteomics profiles. Similarly, a 42 kDa chitin-binding proline-rich protein was another upregulated protein in treatments exposed to nZnO, which is involved in plant-pathogen interaction (Salehi et al., 2021b). The limiting effects of nZnO on protein synthesis were observed at the highest applied dose (Figure 8). Moreover, the SDS-PAGE of this concentration was representative of reduced total protein as depicted in the intensity and width of bands (h pit, 2,000 mg L^–1^). These results confirmed the phytotoxicity of nZnO on the productivity of bean plants at a concentration of less than 1,000 mg L^–1^. As shown in Figure 7, the protein content changes in ZnSO_4_ treatments were significant at *p* ≤ 0.05 as the total protein seems to increase. However, there were no tangible SDS-PAGE protein pattern changes in ZnSO_4_ treatments.

## Discussion

The progressing trend of foliar application of NPs agrees on their phytotoxicity at a higher dosage while observing a positive response on the growth at a lower level. A wide range of effects, from phenotypic symptoms to physiological and biochemical alternations involved in primary and secondary metabolism, have been reported (Salehi et al., 2018, 2020, 2021b; Sharifan et al., 2019). These alternations are often accompanied by plant yield and productivity (Gomez et al., 2020; Sharifan et al., 2020a). Our previous study has already shown both positive and negative effects of nZnO and ZnSO_4_ on bean plant metabolism (Salehi et al., 2021b). In this study, we propounded another aspect of NPs-induced changes in *P. vulgaris* besides the aforementioned reports. Reproductive development in some crops is considered the most stress-sensitive stage after germination-related traits. Therefore, the risk of damage to this period of plant development is often critical (Ma et al., 2020b). The evaluation of male and female gametophytes showed that the development of these reproductive gametes is affected at specific stages, which could be considered as the specific sensitive stages to NPs treatments that seem distinctive from other materials in the micro style. These differences could be due to various factors, including smaller size, higher area surface, lower solubility, and controlled release systems of NPs forms (Khan et al., 2019).

Our results showed that each applied Zn species (NPs and ions forms) might induce different responses corresponding to certain stages of microsporogenesis and macrosporogenesis, which could appear as toxicity symptoms. From the point of the male development, the results confirmed higher toxicity of nZnO compared with their ionic counterpart. This difference is probably due to the physicochemical features of NPs and their ability to cross through plant barriers (Rossi et al., 2019; Sharifan et al., 2020b) and leads to the higher accumulation of ions in tissues. In this term, our previous study showed that higher amounts of Zn were accumulated in the plants exposed to nZnO compared with Zn ions (Salehi et al., 2021b), which could be a critical factor involved in the higher adverse effects on microsporogenesis processes due to the infirmity of performance of the bean plants during the vegetative stage.

The proper male gametogenesis (pollen grain development) is fundamental to plant sexual reproduction. To gain this importance, the main crucial steps in the meiotic cell cycle must be driven forward properly (Coimbra et al., 2009). The higher destructive effects of nZnO likely observed through early phases of meiosis and also the disintegration of tapetal cells caused abnormal PMC, tetrads, and finally, non-functional mature pollen grains compared with ion forms. This timely phenomenon is critical for proper microspore development and pollen maturation. In fact, the disordering of the function of tapetum cell may represent less sporopollenin production or its defective transmit to the cell wall, resulting in inhibition of generating exine and causing cell wall instability (Ariizumi and Toriyama, 2011). Therefore, defects and aberrations in the timing of tapetum development, including hypertrophy and premature as well as delayed degeneration, have been reported, which negatively affect the pollen number and viability (Harsant et al., 2013; De Storme and Geelen, 2014).

Since the tapetum cells produce some necessary metabolites for microspores, they serve as an essential nutrient supplier and provide energy for microspore development (Lei and Liu, 2020), and the interaction between the tapetum layer behavior and normal pollen development is inevitable. Similar results of premature or delayed degradation of the tapetum have been reported in several studies (Vizcay-Barrena and Wilson, 2006; Liu et al., 2018). Additionally, pollen morphology and architecture and its chemical composition were also affected, such as accumulating the starch. Similar findings have previously reported that micro and nano-based metals can be phytotoxic to reproductive organs like reduced pollen germination, pollen tube growth, and minimize the viability of pollen grains as fertilization tools (Kumbhakar et al., 2016; Marmiroli et al., 2021; Yoshihara et al., 2021). For instance, a decreased germination rate and tube elongation of pollen grains (*Lilium longiflorum*) exposed to 100 mg L^–1^ nZnO was observed in the *in vitro* condition (Yoshihara et al., 2021). The aforementioned modifications could alter pollen fitness by reducing the cytoplasmic content and essential materials for exine and intine cell wall production. The literature declared that most of the abnormalities in pollen development are associated with an abnormal tapetal function (Deng et al., 2018; Chaban et al., 2020). As expected, diverse factors such as abiotic stresses influence different developmental stages of pollen grain (Barnabás et al., 2008; Smith and Zhao, 2016). However, there is a remarkable gap in information about the potential effects of NPs on the reproductive developmental stage, in particular, the processes of pollen formation and maturation. Previous studies investigated the developmental fitness under some other environmental risks, in which pollen viability is linked to dehydration and the composition of pollen cytoplasm (Firon et al., 2012; Pacini and Dolferus, 2019). A study showed that *Pennisetum typhoides* pollens containing 5% sucrose exhibited a higher level of non-viability than those containing 14% sucrose, likely due to the high dehydration rate (Hoekstra et al., 1989). Cellular tonicity plays a crucial role in pollen viability, specifically in the productivity of cereals. For example, water stress imposes detrimental effects on the pollen viability, abnormal exine, and intine layer (Pacini and Dolferus, 2019). In agreement with our previous report (Salehi et al., 2021b), the lower relative water content (RWC) in leaves of plants exposed to nZnO during vegetative stages may explain dehydration of pollen grains in the reproductive stage. Therefore, the observed unbalance fitness in pollen grains could be due to physiochemical alternations and also cytological aberrations during meiosis and mitosis processes, resulting in reduced pollen viability and consequently a higher mortality rate of seeds. In agreement with this, Marmiroli et al. (2021) has recently reported some regulated pollen-specific genes in zucchini (*Cucurbita pepo* L.) plants upon exposure to CuO nanoparticles. This study, along with data presented in this study, would strengthen the concept of NPs translocation into the floral parts and also trigger an NPs-specific response that has been proven somehow distinct from that induced by other counterparts (such as bulk and ion forms). Moreover, it has been shown that those genes related to cell wall composition, dynamic 3-dimensional structures (which fill the cytoplasm), and starch metabolism were the targets of NPs (Marmiroli et al., 2021). All of these abovementioned metabolic and genomic pathways are considered key aspects of pollen maturation.

Megasporogenesis (female gametophyte development) in the ovule of a flowering plant serves as the site of double fertilization (which later develops into the embryo and endosperm). This phenomenon is crucial for plant reproduction and world food production. Despite that, only a few reports are available on female gametophyte development under diverse environmental stresses (Klatt et al., 2016). Likewise, the number of studies upon exposure to NPs is almost zero. For example, a few studies have shown several abnormalities like reduced ovule size and its viability upon cold stress [reviewed by Zinn et al. (2010)]. In this trial, apoptosis of embryo sac cells, including egg cells and synergid cells, disturbing the endosperm division, embryonic tissues damage, and embryo abortion were among the traceable modifications. Therefore, the female gametophyte may not be affected as intensely as male development by such abiotic stresses. Nevertheless, given the interaction between these two reproductive constituents and the necessity of this interaction for pollen tube growth and guidance through the pistil, any damages to these reproductive cells could affect the fertilization (Klatt et al., 2016). For example, in our experiment, apoptosis of synergid cells may disturb the precise track of pollen tube growth to central cells and egg cells, leading to produce unfertilized seeds as observed at higher dosages of Zn treatments. Overproduction of ROS most likely causes the embryo-sac cellular apoptosis and oxidative stress induced by NPs (Rossi et al., 2018; Salehi et al., 2021b). The nZnO used in this study at higher concentrations have led to the upregulation of several proteins (i.e., peroxiredoxin, non-specific lipid transfer protein, and wound-induced basic protein) and metabolites (glucosinolates and pteridine-based metabolites), most likely due to response to oxidative stress in the leaves of exposed plants (Salehi et al., 2021b). The previously published data showed that exposure of bean plants to Zn species, especially nZnO, resulted in cumulative effects of ROS accumulation leading to the reduced level of antioxidant enzymes such as catalase (CAT), peroxidase (POX), and ascorbate peroxidase (APX). As a result, impaired male and female development is expected, such as premature pollen abortion, non-functional pollens, cellular apoptosis, and seed abortion.

Zinc deficiency has been associated with pollen infertility and reduced seed productivity in wheat and lentil (Pandey et al., 2006; Cakmak, 2008; Zulfiqar et al., 2020). Unlike the high-level NPs (>1,000 mg L^–1^), at lower levels, no visible toxic symptoms on cells or tissues of reproductive organs were observed. Nevertheless, Zn ions at low concentrations elevated the total seed yield and promoted the production of healthier seeds. It has been proven that Zn drives many biological mechanisms, including pollen development and seed yield. In this regard, our results suggested stimulating effects of Zn ions at low amounts on sporogenesis, gametogenesis, and reproduction. Previous studies have confirmed the plant uptake and distribution of Zn species either in the form of NPs or ions, which impact developmental stages from germination to seed production (Wang et al., 2018; Ma et al., 2020a,b). However, few studies have shown the positive or negative effects of nZnO on seed productivity. For example, the soybean plants exposed to 500 mg kg^–1^ ZnO NPs did not produce seeds (Yoon et al., 2013). In contrast, adverse effects on the yield of sweet potato (*Ipomoea batatas* L.) were only observed at 1,000 mg kg^–1^ nZnO concentration (Bradfield et al., 2017). Therefore, further studies are needed to elucidate their potential effects on the storage components of both pollen grains and seeds.

## Conclusion and Future Perspective

Given the critical role of Zn in entire plant development, there has been remarkable interest in its nanoscaled form application as a slow-release fertilizer. However, despite its proven phytotoxicity at the vegetative stage of development, there is no information on the reproductive perspective. Therefore, apart from the previous reports, this study investigated the impacts of foliar application of nZnO on plant reproductive development. Both male and female organs have been monitored from the earliest emergence until full maturity. The spray application of Zn ions at lower levels induced positive responses on sporogenesis and gametogenesis processes of male and female organs of bean plants, resulting in higher seed yield. However, nZnO impaired the normal developmental trend; particularly, two developmental processes (i.e., meiosis and pollen development) were distinctively affected depending on NPs dosages. In terms of male development, it was indicated that the most induced alternations occurred during microspore development. Moreover, tapetum dysfunction appears to be a critical factor involved in premature microspore and pollen abortion. The impaired reproductive and reduced seed productivity with higher NPs levels may be explained by a higher accumulation of Zn and reactivity in this form, oxidative stress induced by NPs, interrupting the pollen performance, and physiochemistry. In fact, increased non-viability of pollen was attributed to structural-induced defects during microgametogenesis. Overall, the results confirm that nZnO exerted stronger phytotoxicity on reproductive components fitness while an ion at a certain amount positively influences plant development and could lead to a higher yield. Since there is a complicated network between vegetative and generative stages of plant growth, a comprehensive omics analysis in both stages might uncover more mechanistically the distinct response of nZnO than its counterparts. Therefore, there is an urgent need to elucidate the underlying molecular mechanisms of nZnO and other nano-based materials on reproductive organs, especially pollen grains, and their safety in agro-sustainable application.

## Data Availability Statement

The original contributions presented in the study are included in the article/Supplementary Material, further inquiries can be directed to the corresponding author.

## Author Contributions

HS and ACR conceived the idea. HS performed the work, analyzed the data, and wrote the manuscript. AR, HSh, and ACR reviewed and edited the first draft. ACR and RKV proofread and edited the manuscript. All authors have read and agreed to the published version of the manuscript.

## Conflict of Interest

The authors declare that the research was conducted in the absence of any commercial or financial relationships that could be construed as a potential conflict of interest.

## Publisher’s Note

All claims expressed in this article are solely those of the authors and do not necessarily represent those of their affiliated organizations, or those of the publisher, the editors and the reviewers. Any product that may be evaluated in this article, or claim that may be made by its manufacturer, is not guaranteed or endorsed by the publisher.
