# Supplementary material for: Aerially Applied Zinc Oxide Nanoparticle Affects Reproductive Components and Seed Quality in Fully Grown Bean Plants (Phaseolus vulgaris L.)
Source: Front Plant Sci. 2022 Jan 12;12:808141. doi: 10.3389/fpls.2021.808141 (PMC8790032; doi:10.3389/fpls.2021.808141)
Supplement: Supplementary file 1 [file Data_Sheet_1.docx]

**Supplementary Information**


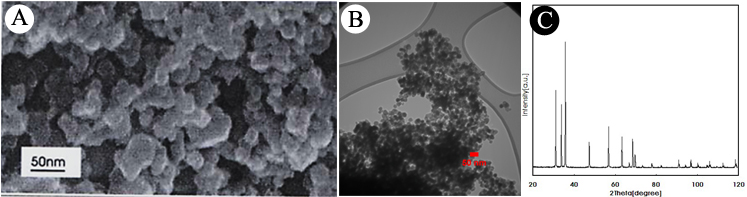
**Supplementary Figure 1.** (A) SEM image at high magnification and (B) TEM image at low magnification of nZnO.

Both optical techniques were used to characterize the nanoparticles for their dominant size and morphology.


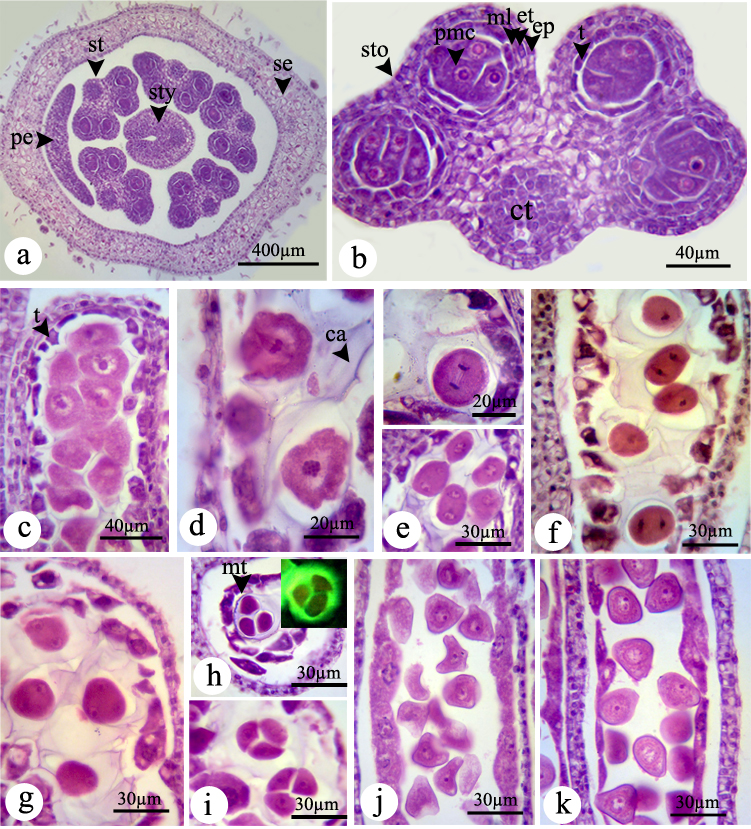


**Supplementary Figure 2.** Microsporogenesis of *Phaseolus vulgaris* in normal condition. a) Transverse section of young anther showing five stamens accompanied with unilocular ovary. b) Transverse section of four-locule anther composed of wall layers (epidermis, endothecium, middle layer, and tapetum) and pollen mother cells. c) Longitudinal section of anther showing the initiating of meiosis in pollen mother cells (Prophase I). d) Metaphase I. e) Anaphase I (up) and telophase I (down). f) Metaphase II. g) Telophase II. h) Tetrad formation with callose layer around it. i) The initiation of releasing microspores from tetrad. j) Released microspore with irregular shapes. k) Immature pollen grains with regular shapes. Abbreviations: Connection tissue (ct), endothecium (et), epidermis (ep), middle layer (ml), microspore tetrads (mt), petal (pe), pollen mother cells (pmc), sepal (se), stamen (st), stomium (sto), style (sty), tapetum layer (t), callose (ca).


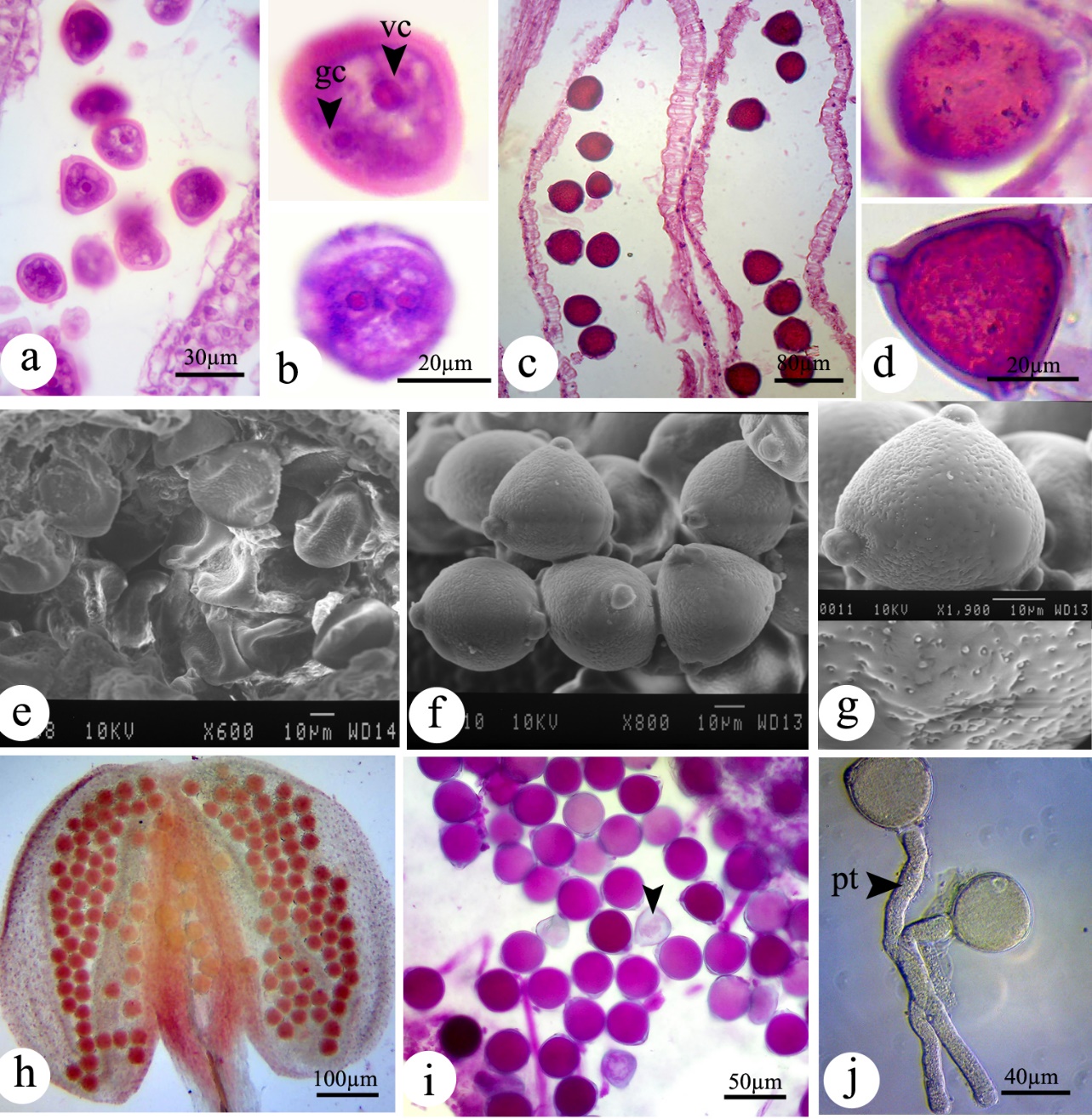


**Supplementary Figure 3.** Continue of microsporogenesis and male gametophyte formation of *Phaseolus vulgaris* in normal condition. a) Two- nucleated pollen grains. b) Generative and vegetative nuclei. c) Anther loculus. d) Mature pollen grain with accumulated starch. e) Scanning electron microscopy image of the immature pollen grain. f, g ) SEM images of the mature pollen grain. h) The staining of anther and pollen grains using acetocarmine. i) Alexander staining of pollen grains, viable pollen grains are purple, and non-viable is colorless. j) Pollen grain and generating pollen tube. Abbreviations: Generative cell (gc), vegetative cell (vc), pollen tube (pt).


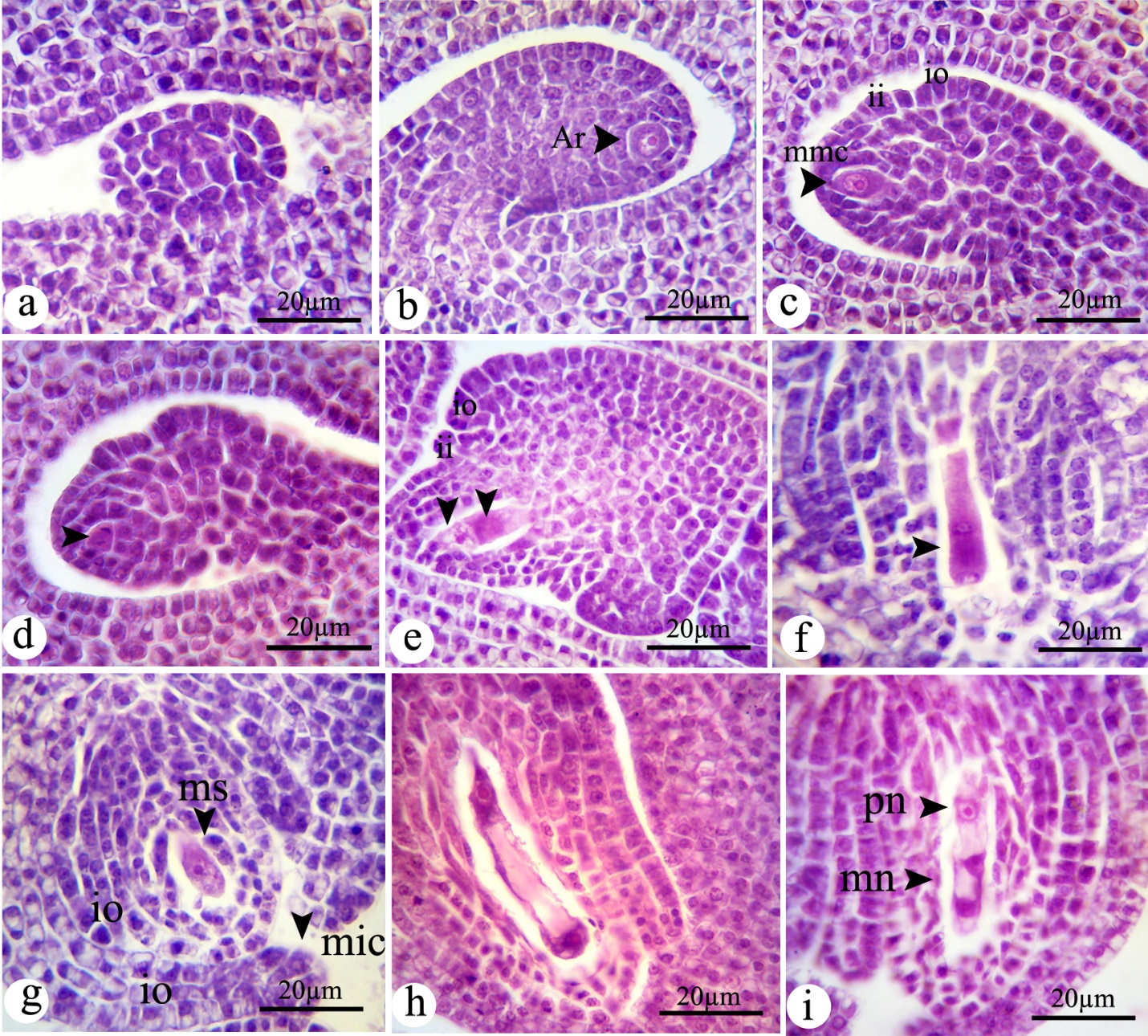


**Supplementary Figure 4.** The development of female gametophyte in *Phaseolus vulgaris* in normal condition. a) The initiation of ovule primordium. b) The differentiation of archespores cell. c) Megaspore mother cell at prophase stage. d) Megaspore mother cell at anaphase I stage. e) Megaspore mother cell at telophase I stage and dyad formation (arrows). f) Showing the formation of a linear tetrad (arrow). g) Functional megaspore and the initiation of one-nuclear embryo sac. h) Two-nucleated embryo sac. i) Four-nucleated embryo sac. Archespore (Ar), outer integument (io), internal integument (ii), megaspore mother cell (mmc), micropyle nuclei (mn), polar nucleus (pn), megaspore (ms), micropyle (mic). All sections are longitudinal.


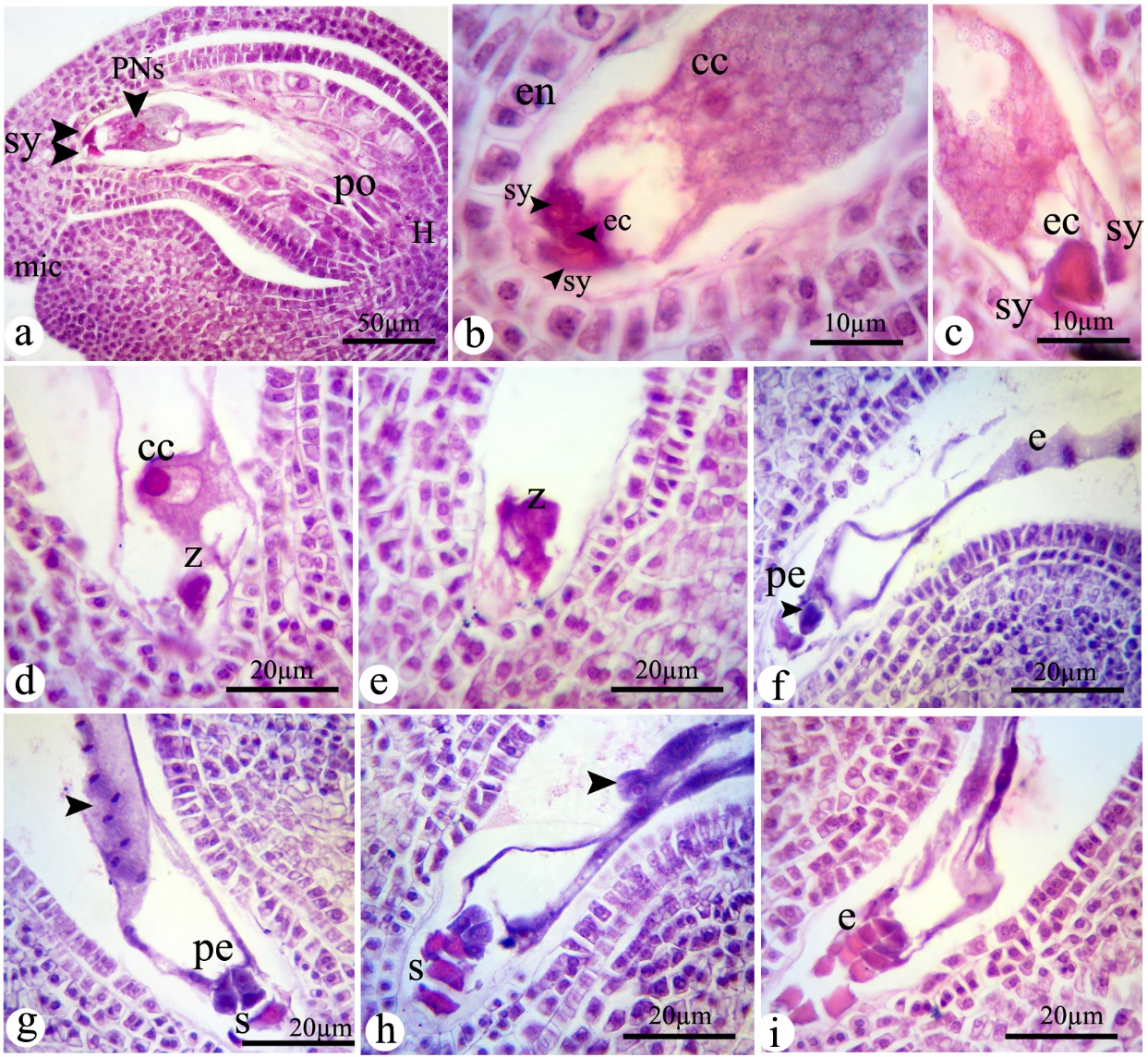


**Supplementary Figure 5.** The development of the embryo sac and initiating the embryo. a) Eight-nucleated embryo sac containing two synergids, polar nuclei, hypostase, and postament tissues. b) Egg apparatus composed of an egg cell, synergids, polar cells, and central cell formation. c) Degenerating of synergids and the contiguity of the sperm cell and egg cell. d) Zygote cell formation. e) Zygote with short suspensor. f) Two-celled pre-embryo and the metaphase division of endosperm. g) Four-celled pre-embryo and the anaphase mitotic division of endosperm. h) The division of suspensor cells, endosperm at the interphase stage of mitosis. i) Eight-celled embryo. All sections are longitudinal. Synergids (sy), polar nuclei (PNs), postament (po), hypostase (H), egg cell (ec), central cell nucleus (cc), endothelium (en), zygote (z), pre-embryo (pe), embryo (e), suspensor (s).

**
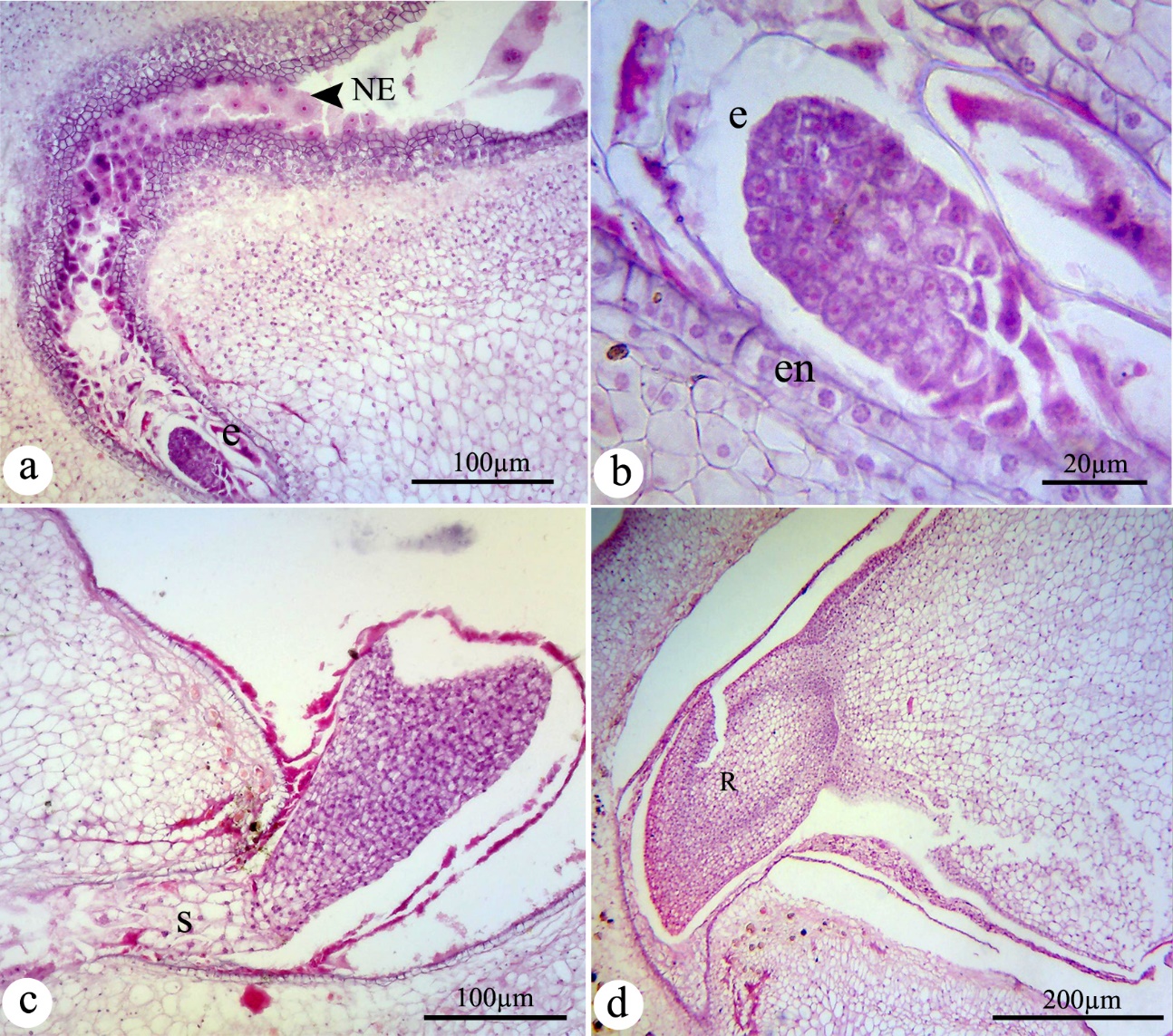
**

**Supplementary Figure 6.** Embryo development. a, b) 32-cellular embryo (torpedo-shaped) and nuclear endosperm, c) Heart-shaped embryo, and elongation of the suspensor. d) Development of cotyledons. All sections are longitudinal. Embryo (e), endothelium (en), nuclear endosperm (EN), suspensor (s), radicle (R).
